# Dendritic Cells and Their Role in Immunotherapy

**DOI:** 10.3389/fimmu.2020.00924

**Published:** 2020-05-21

**Authors:** Alycia Gardner, Álvaro de Mingo Pulido, Brian Ruffell

**Affiliations:** ^1^Department of Immunology, H. Lee Moffitt Cancer Center and Research Institute, Tampa, FL, United States; ^2^Cancer Biology PhD Program, University of South Florida, Tampa, FL, United States; ^3^Department of Breast Oncology, H. Lee Moffitt Cancer Center and Research Institute, Tampa, FL, United States

**Keywords:** dendritic cells, immunotherapy, immune checkpoint blockade, vaccines, cancer

## Abstract

Despite significant advances in the field of cancer immunotherapy, the majority of patients still do not benefit from treatment and must rely on traditional therapies. Dendritic cells have long been a focus of cancer immunotherapy due to their role in inducing protective adaptive immunity, but cancer vaccines have shown limited efficacy in the past. With the advent of immune checkpoint blockade and the ability to identify patient-specific neoantigens, new vaccines, and combinatorial therapies are being evaluated in the clinic. Dendritic cells are also emerging as critical regulators of the immune response within tumors. Understanding how to augment the function of these intratumoral dendritic cells could offer new approaches to enhance immunotherapy, in addition to improving the cytotoxic and targeted therapies that are partially dependent upon a robust immune response for their efficacy. Here we will discuss the role of specific dendritic cell subsets in regulating the anti-tumor immune response, as well as the current status of dendritic cell-based immunotherapies, in order to provide an overview for future lines of research and clinical trials.

## Introduction

Immunotherapy has revolutionized the treatment of many solid and hematological malignancies, with immune checkpoint blockade (ICB), adoptive cell therapy (ACT) using tumor infiltrating leukocytes (TIL), and vaccine strategies targeting different aspects of the immune-oncology cycle to improve the functionality of T lymphocytes. Each of these strategies, however, is necessarily predicated on the initiation of the cycle, namely the presentation of tumor antigens by professional antigen-presenting cells (APCs) (1). APCs can be defined by their ability to capture, process, and present exogenous antigen to T cells, and are usually identified by their constitutive expression of major histocompatibility complex (MHC) II and costimulatory molecules. Thus, dendritic cells (DCs), macrophages, and B cells are normally considered to be the three major populations of APCs. It should be noted that other populations also constitutively express MHCII, including thymic epithelial cells, while still others can acquire exogenous antigen, and express MHCII following activation, including eosinophils and basophils (2, 3). However, in the context of solid tumors, antigen uptake, and presentation are primarily the domain of macrophages and DCs (4). While macrophages are the dominant phagocytic population in tumors, they do not migrate to the lymph nodes and are unable to activate T cells *ex vivo* (4). Instead, macrophages are usually found to blunt T cell responses against tumors via multiple mechanisms and act to suppress therapeutic response to ICB as well as chemotherapy and irradiation (5, 6). DCs thus have a unique ability to transport tumor antigen to the draining lymph nodes to initiate T cell activation, a process that is required for T cell-dependent immunity and response to ICB (4, 7–10). Tumor-resident DCs also have an emerging role in regulating the T cell response within tumors during therapy (4, 11–14). These functions place DCs at the fulcrum of the anti-tumor T cell response and suggest that regulating the biological activity of these cells is a viable therapeutic approach to indirectly promote a T cell response during therapy.

## Dendritic Cells in Cancer

DCs are the quintessential APCs of the immune system, responsible for bridging the gap between innate and adaptive immunity, including the activation of anti-tumor T cells (4, 7–10). DCs arise from bone marrow progenitors known as common myeloid progenitors (CMPs). From here, two cell subtypes diverge. Expression of the transcription factor Nur77 drives the differentiation of CMPs into monocytes, which can further differentiate into monocyte DCs (moDCs) under inflammatory conditions (15–18). In the absence of Nur77, CMPs differentiate into the common dendritic cell progenitor (CDP), which gives rise both to plasmacytoid DCs (pDCs) and conventional DCs (cDCs) (15). Differentiated cDCs are initially immature, requiring maturation signals (for instance, damage or pathogen associated molecular patterns [DAMPs or PAMPs], or inflammatory cytokines) to fully effect their role in the immune response (15, 18). Upon maturation and activation, DCs downregulate phagocytosis, increase MHC and costimulatory molecule expression, increase cytokine production, and display enhanced migration to lymph nodes, likely driven by higher expression of C-C chemokine receptor 7 (CCR7) (15). As a result of the phenotypic changes that occur during activation, mature DCs are able to prime naïve T cells and initiate the adaptive immune response.

cDCs can be further divided into two subsets, known as type one (cDC1) and type two (cDC2) conventional DCs. cDC1 are defined by reliance on the transcription factors BATF3 and IRF8 for development, and express several common surface markers across species, including XCR1, CLEC9A, CADM1, BTLA, and CD26 (19). However, the cells were originally identified by surface expression of CD8α (lymphoid organ resident) or CD103 (peripheral tissue resident) in mice (20–22) and CD141 (BDCA-3) in humans (23–25), making these the most commonly used markers. In both organisms, the cDC1 subset displays enhanced ability to cross-present exogenous antigen and activate CD8^+^ T cells (15, 18, 26), but this functional demarcation between the cDC1 and cDC2 subset is more pronounced in mice than in humans (19). In both mice and humans cDC1s represent a small percentage of immune cells in circulation. cDC1 accounted for <0.01% of CD45^+^ cells in the blood of healthy human donors, as well as <0.1% of CD45^+^ cells in surveyed tissue sites (27).

cDC2 are easiest to identify by the absence of cDC1 markers, but higher expression of CD11b, CD1c, and SIRPα (CD172α) is also frequently used to distinguish the population, with IRF4 acting as the key transcription factor (28–31). No specific markers identify migratory from resident cDC2 populations in mice, but differential expression of CD11c and MHCII can be used as a distinguishing feature (15). In mice, cDC2 are primarily responsible for presentation of endogenous antigen to CD4^+^ T cells and shaping the resulting polarization of the cells, with the ability to polarize CD4^+^ T cells also observed with human cDC2 (32). As mentioned, however, human cDC2s can cross-present antigen and produce high levels of interleukin (IL)12, properties that are largely restricted to the cDC1 subset in mice (19). Thus, despite the critical role of cDC1s in the development and maintenance of anti-tumor immunity in experimental models (15), it is possible cDC2s have an unidentified role in human cancers. Indeed, a recent study demonstrated a correlation between cDC2 abundance and non-T_reg_ CD4^+^ T cell infiltration into head and neck squamous carcinomas. High cDC2 and low T_reg_ infiltration was also associated with longer progression-free survival (33).

### Type 1 Conventional DCs

In mice, cDC1 are responsible for the induction of the “cancer-immune cycle,” as *Batf3-*deficient mice are unable to reject even highly immunogenic tumors or respond to immune-mediated therapies such as checkpoint blockade and adoptive T cell transfer (7–10, 13, 34). This has been traced to the ability of cDC1s to transport antigen from tumors into draining lymph nodes, with migratory cDC1s being the only APC subset capable of causing robust activation and proliferation of CD8^+^ T cells *ex vivo* (9, 10). Additionally, migratory cDC1 represented the only cDC subset able to transport antigen to the lymph node in two studies using melanoma models (9, 10). cDC trafficking to the lymph node and generation of a systemic anti-tumor immune response is governed by CCR7 expression (9). Mice lacking CCR7-expressing cDC1 failed to recruit CD8^+^ T cells to the tumor, and the T cells that were present in the tumor microenvironment failed to proliferate, leading to an overall lack of immune control (9). Similarly, the inability of tumors to recruit the cDC1 subset prevents an effective CD8^+^ T cell response from developing (35, 36), while increasing the number of cDCs in the tumor can restore response to immunotherapy (10, 35). Taken together, these studies strongly support CD103^+^ migratory cDC1 as critical for the induction of anti-tumor immunity. In non-tumor models of immunity, lymph node-resident cDC also acquire antigen from migratory cDCs and are needed to initiate an optimum CD8^+^ T cell response (37, 38). Whether there are sequential roles for migratory and resident cDC1s during the development of an anti-tumor response is not yet known. However, cross-presentation by cDC1s is critical for the induction of an adaptive immune response by cytotoxic CD8^+^ T cells, with mice specifically deficient in cross-presentation-capable cDC1s unable to reject highly immunogenic fibrosarcoma tumors (39). In addition, cross-presentation by cDC1 is enhanced by type I interferon (IFN) signaling (40). The absence of type I IFN in the tumor microenvironment, or the inability of cDC1 to sense type I IFN, are sufficient to impair the development of a CD8^+^ T cell response (34, 40). Taken together, these studies emphasize the importance of cross-presentation of tumor antigen to naïve CD8^+^ T cells in the lymph node in the induction of a successful anti-tumor immune response.

**Figure 1:**
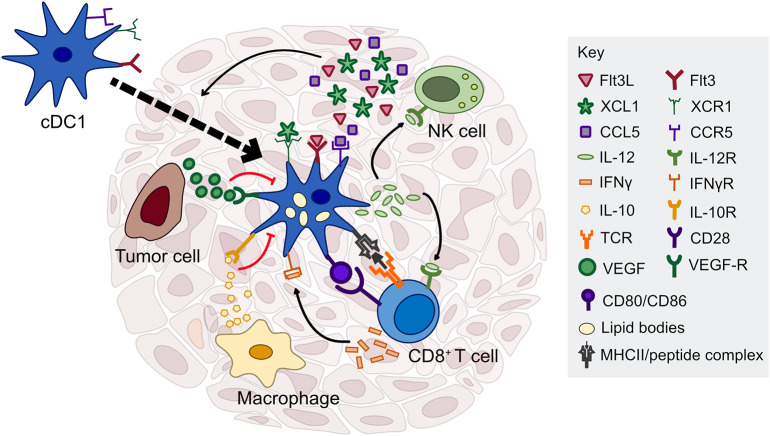
Factors regulating cDC1 function in the tumor microenvironment. cDC1s interact with several immune cell types through cytokine and chemokine signaling, including NK cells, T cells, and macrophages. NK cells are critical for cDC1 recruitment and survival in the tumor through production of Flt3L, CCL5, and XCL1. cDC1 have the capacity to cross-present exogenous antigen to CD8^+^ T cells and stimulate naïve and previously activated T cells *ex vivo*; however, the importance of antigen presentation by cDC1s in the tumor microenvironment is currently unclear. In contrast, cDC1 production of IL-12, driven by IFN-γ or other inflammatory mediators, is necessary to sustain a T cell response during chemotherapy or immune checkpoint blockade. cDC1 production of IL-12 can be directly inhibited by IL-10 released by macrophages or other immunosuppressive cells, as well as tumor-derived factors that inhibit the maturation of cDC1s such as VEGF.

It is also becoming increasingly clear that cDC1s have a critical role in maintaining CD8^+^ T cell function within tumors. In secondary lymphoid organs and in non-tumor models of immunity, the organization of immune cells is critical for effective signaling (41, 42). The localization of T cells near cDCs, especially, has been shown to be critical to the induction of an adaptive immune response (43, 44). Consistent with this, cytokine production by tumor cDC1s has proven essential for immunotherapy. In the of context adoptive cell therapy (ACT), efficacy required cDCs capable of CXCL9/CXCL10 production in order to drive tumor infiltration by the transferred T cells (13). cDC1 production of CXCL9/CXCL10 and expression of the cognate receptor, CXCR3, on CD8^+^ T cells, has also recently been shown to be critical for response to anti-PD-1 or anti-TIM-3 therapy (11, 14). Surprisingly however, this was not mediated by increased CD8^+^ T cell tumor infiltration, but rather enhanced effector function in endogenous CD8^+^ T cells. How chemokine expression by tumor cDC1s promotes a T cell response is unclear, but may relate to cDC1s being largely responsible for production of IL-12 within tumors (4, 45). In support of this, cDC1 production of IL-12 was found to induce IFNγ production by CD8^+^ T cells following PD-L1 blockade, and the feedback loop between IL-12-producing cDC1s and IFNγ-producing CD8^+^ T cells was necessary for therapeutic efficacy (12). Similarly, IL-12, CXCL9/10, and IFNγ are all required for response to the combination of paclitaxel chemotherapy and TIM-3 blockade (11). Taken together, the data indicate the importance of cDC1 and CD8^+^ T cell crosstalk in the tumor microenvironment and suggest that targeting this interaction is therapeutically viable (Figure 1). Interestingly, a recently published study used single cell RNA sequencing (scRNA-seq) to identify a subset of regulatory DCs in lung tumors (46). Although these were shown to arise from both the cDC1 and cDC2 lineage following maturation and uptake of tumor antigen, the authors specifically focused on the regulatory DCs of the cDC1 lineage, and showed that blockade of IL-4 could reestablish IL-12 expression, thus improving CD8^+^ T cell function and tumor control (46).

Another recent advancement in the field is the characterization of natural killer (NK) cell and cDC1 interplay within tumors. Two groups independently showed that NK cell production of either FMS-related tyrosine kinase 3 ligand (Flt3L) or CCL5 and XCL1 induces cDC1 recruitment into the tumor microenvironment (36, 47). Analyses of gene signatures in human tumors indicate that the presence of NK cells correlates with the presence of cDC1 in this context as well, suggesting that manipulation of NK cell presence within the tumor could indirectly improve the adaptive immune response (36, 47). Communication in the opposite direction has also been shown to be required, with IL-12 production by cDC1 leading to IFNγ production by NK cells (48). Neutralization of IL-12 or the absence of cDC1 in *Batf3*-deficient mice increased lung colonization following tail-vein injection of multiple tumor cell lines (48). The requirement for cross-talk between cDC1 and multiple immune subtypes is indicative of the complexity of the immune response within the tumor and suggests that the localization of leukocytes within the tumor is a critical regulator of their function. Improvements in imaging techniques and analysis platforms will help dissect some of this complexity.

At both the genetic and functional level, human cDC1 show similar characteristics to mouse cDC1 (25, 31), suggesting that mouse models to study cDC1 function will be informative in translating the biology to the context of humans. In particular, a recently published study used scRNA-seq to profile myeloid populations in human and mouse lung cancers, and found a high degree of concordance between DC subsets in the two species, including cDC1 (31). The same study assessed the association of the gene signatures most specific to individual cell types and compared them with patient prognosis. cDC1 genes were generally found to be associated with positive prognosis, suggesting that the presence of cDC1 in human lung tumors is associated with better survival (31). Similar findings have been made in hepatocellular carcinoma (49), and the presence of DCs in breast tumors (11), along with the ratio of CD103^+^ cDC1 to CD103^−^ DCs in breast cancer, head and neck squamous cell carcinoma (HNSCC), and lung adenocarcinoma (4), have all been shown to correlate with improved patient prognosis. In addition, the presence of cDC1 within human melanoma tumors correlated with improved response to anti-PD-1 therapy (36) as well as with higher CD8^+^ T cell infiltration into tumors (33), which is associated with a positive prognosis across multiple tumor types (50). Furthermore, genes specific for cDC1 correlate with the presence of *CXCL9* expression by human tumors in the TCGA database (11, 13), and cDC1 in human breast tumors exhibit expression of CXCL9 by immunofluorescence (11), further indicating that human cDC1 are likely to produce similar chemokines and play a similar role in the tumor microenvironment as mouse cDC1. As *CXCL9* expression also correlates with response to anti-PD-1 (14), there is likely a critical role for cDC1s in the context of patient response to ICB as well, although this has not been directly tested.

### Type 2 Conventional DCs

While the aforementioned data suggest that cDC1 may be the only DC subset required for the induction of anti-tumor immunity, this neglects the importance of CD4^+^ T cells, which play a critical role in supporting CD8^+^ T cell activity (suggesting a role for cDC2 antigen presentation to CD4^+^ T cells) (51–54). While cDC2 are dispensable for CD8^+^ T cell activation and proliferation in some tumors (4, 9), this may be due to the specific models and therapies examined. For example, cDC2s were found to be important during response to anthracycline chemotherapy (55), and certain tumor models are responsive to adoptively transferred CD4^+^ T cells (56). There are also several reports describing recognition of tumor antigens by human CD4^+^ T cells (56). As with cDC1, scRNAseq has shown that at the genetic level, mouse and human cDC2 subsets in lung tumors show a high degree of overlap (31). This includes the existence of functionally distinct subsets marked by expression of T-bet and RORγt (57). Additionally, it was recently shown that following depletion of regulatory T cells (T_reg_), a subset of cDC2 can effectively elicit intratumoral CD4^+^ T cell responses and subsequent tumor control in a mouse model of melanoma (33). Upon T_reg_ depletion, cDC2 were able to migrate to the draining lymph node and effectively induce differentiation of conventional CD4^+^ T cells (33). The observed increase in tumor rejection specifically required CD4^+^ T cell priming in the lymph node, as FTY720 blockade of lymph node egress prevented the anti-tumor immune response (33).

Interestingly, when the cDC2 gene signature was correlated with prognosis for lung adenocarcinoma patients, cDC2 were the DC subset most strongly associated with a positive prognosis (31). Similarly, high levels of cDC2 in HNSCC and melanoma tumors, when combined with low levels of regulatory T cells, correlated with longer progression free survival and higher levels of CD4^+^ T cell infiltration, further suggesting a role for both cDC2 and CD4^+^ T cells in human tumors (33). A substantial degree of heterogeneity in the cDC2 subset isolated from draining lymph nodes of human melanoma patients also correlates with the heterogeneity observed in cDC2 isolated from mouse tumors, with similar characteristics observed in both subsets (33). Given these data, it will be interesting to examine whether T_reg_ are also preventing cDC2 function in contexts other than melanoma, and whether depletion of the T_reg_ may augment the anti-tumor immune response in human tumors via increased cDC2 and CD4^+^ T cell activity.

### Plasmacytoid Dendritic Cells

In contrast to cDCs, whose role in anti-tumor immunity is associated with antigen presentation, plasmacytoid DCs (pDCs) are usually associated with response to viral RNA and DNA via production of high levels of type I IFN, along with other inflammatory cytokines such as IL6 and TNFα. However, pDCs do express MHCII and costimulatory molecules and could therefore potentially act as antigen-presenting cells, although the antigen processing capabilities of the cells are unclear (18, 58). Interestingly, pDCs differentiate from myeloid CDP as well as from IL-7R^+^ lymphoid progenitors (59), resulting in cells that are phenotypically similar but with distinct functional capacities (59). Specifically, only myeloid-derived pDCs were found to process and display antigen (59). The role of pDCs in cancer may therefore depend upon the extent to which they are myeloid derived, in addition to their activation state. At least one study has shown that tumor-associated pDCs are largely inert, but that following intratumoral injection of a TLR7 ligand, pDCs can induce anti-tumor immune responses (60). Whether this response is directly attributable to antigen presentation by myeloid-derived pDCs or is a result of type I IFN activation of cDC function is less clear (61).

In a similar vein, the role of pDCs in human tumors is less established than that of the cDC subsets. As with cDC1 and cDC2, scRNAseq indicates that the human pDCs mirror mouse pDCs (31). The human pDC gene signature also correlates with a positive prognosis in lung adenocarcinoma, although to a lesser degree than either cDC1 or cDC2 (31). In contrast, the presence of pDC in breast tumors, as assessed by immunohistochemical staining, strongly correlated with a poor overall prognosis (62). Additionally, pDCs found in the ascites of patients with ovarian carcinoma induced IL-10-producing CD8^+^ regulatory T cells and inhibited T cell proliferation (63). High-dimensional analysis has recently been employed by several groups to identify heterogeneity within the classically defined pDC population in human samples (64–66), raising the possibility that the conflicting roles of pDCs in human tumors could be attributed to the conflation of multiple subsets.

### Monocyte Dendritic Cells

Monocyte-derived DCs (moDCs) differentiate from Ly6C^+^ or CD14^hi^ monocytes in mice and humans, respectively, generally under inflammatory conditions (19). Identification of moDCs has historically been difficult, as the markers used for identification overlap substantially with those expressed by macrophages and CD11b^+^ DCs in mice. Recently, however, expression of the Fc receptors FcγRI and FcεRI were used to distinguish the subset (67). In contrast to the ability of cDCs to present antigen to T cells, moDCs have not been shown to transport antigen to the lymph nodes and activate T cells. As a result, it is unclear what role moDCs can have in inducing a *de novo* T cell response. However, the recruitment of moDCs is enhanced under inflammatory conditions, which can lead to the induction of “TipDCs” (tumor necrosis factor (TNF) and NOS2-producing inflammatory dendritic cells) from moDCs. It was also recently shown that for mice given adjuvant therapy with polyinosinic:polycytidilic acid (Poly [I:C]), moDCs were required for the anti-tumor response, whereas cDC1 were dispensable (68). moDCs have also been shown to enhance the survival of adoptively transferred T cells (69) and may further regulate T cell activity within tumors through production of TNFα and NOS2 (18). Activation of p53 in myeloid precursors can even promote the formation of CD103^+^ moDCs with the capacity to cross-present antigen and produce high amounts of IL-12 (70). moDCs also appear to play a critical role in the regulation of graft-vs.-leukemia (GVL) responses following therapeutic bone marrow transplants, with inhibition of XBP-1 splicing helping to prevent graft-vs.-host disease while maintaining a GVL response in both murine and human xenograft models (71, 72). Thus, while the role of moDCs in the development of spontaneous anti-tumor immunity is unclear, they appear critical in sustaining an immune response during certain inflammatory conditions.

## Dendritic Cell-Based Therapies

**Figure 2:**
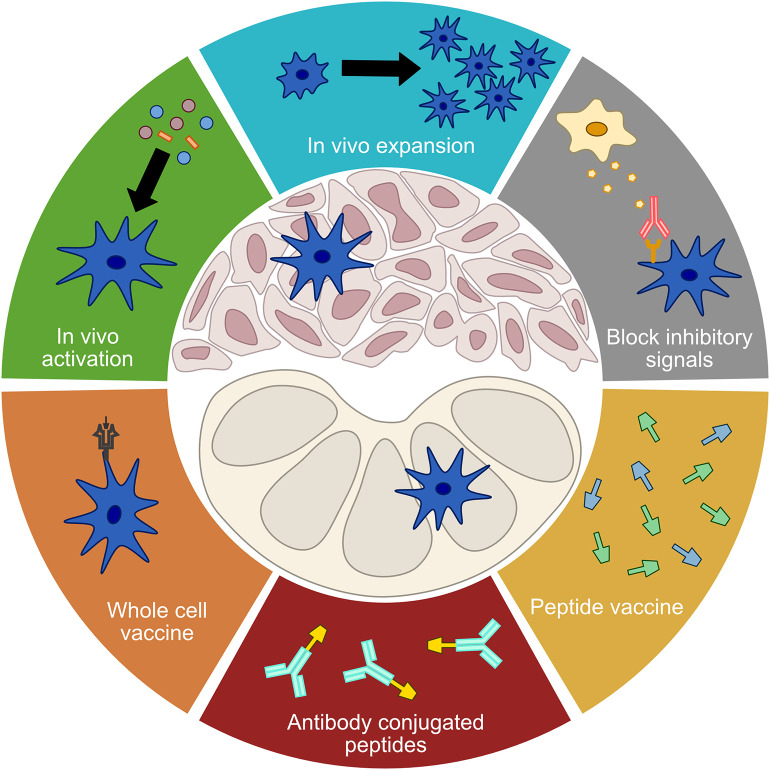
Treatment modalities targeting DCs. A number of current treatment modalities aim to address limited DC functionality in order to elicit or enhance anti-tumor immune responses. Treatments that seek to improve the function of tumor DCs include *in vivo* activation, *in vivo* expansion, and the blocking of inhibitory signals. Vaccination approaches that seek to bypass tumor DCs and directly stimulate a *de novo* T cell response in the lymph nodes include whole cell vaccines, antibody conjugated peptides, and free proteins or peptides.

Immunotherapy continues to represent a promising avenue for new cancer therapies, especially since many patients who respond exhibit durable responses. However, response rates for many tumor types are still low, underscoring the need for continued improvement in our understanding of anti-tumor immunity and approaches to enhance it. As expanded upon in the first section, cDCs are central inducers of the immune response, and targeting them may provide a method of improving immune responses in cases where targeting T cells alone is ineffective. As DCs, especially cDC1, tend to correlate with a positive prognosis when they are present in tumors, therapies targeting DCs focus on enhancing DC function, increasing their numbers, or bypassing the tumor microenvironment to promote systemic *de novo* anti-tumor immunity (Figure 2).

### *In vivo* Activation

One of the earliest approaches to immunotherapy was the attempt to revert suppression of cDCs in the tumor microenvironment by providing exogenous activation signals. Toll-like receptors (TLRs) are one of the major pathogen- and damage-sensing pathways, with 13 different TLRs present in mice (TLR1-TLR13) and the first 10 also present in humans. DCs subsets display differential TLR expression patterns in both species (73, 74). For example, in humans, pDCs preferentially express TLR7 and 9, cDC1 preferentially express TLR3 and 8, and cDC2 preferentially express TLR1 and 6 (73). This means DCs preferentially recognize different pathogenic/danger-associated signals and can be targeted with specific agonists, potentially with the goal of optimally shaping the anti-tumor immune response. However, the identification and therapeutic use of TLR agonists predates the classification of the cDC subsets, and limited work has been done in this area.

In humans, TLR7 and TLR9 are among the more widely explored targets given their capacity for inducing a type I IFN response. Topical TLR7 agonists including imiquimod and R848 have been shown to induce an immune response as well as promote some level of tumor control in a variety of cancer types, including melanoma and breast cancer (75–77). Indeed, a number of clinical trials are currently ongoing to test TLR agonists in breast cancer patients, with one having observed immune-mediated rejection of skin metastases following treatment with imiquimod (75). Topical application carries a clear drawback, in that it can only reasonably be used in situations where either the induction of a systemic immune response will be able to induce tumor control, or where tumors are close enough to the body's surface that a local immune response can be induced. As a result, TLR7 agonists with non-topical application methods are also under development. One such agonist is 852A, which has been shown to induce CXCL10 and IL-1RA production, although minimal tumor control was observed in initial clinical trials (78, 79). In addition to TLR7 activation, DCs can be targeted via TLR9 agonists (73), with activation of TLR9 using CpG oligodeoxynucleotides (ODNs) causing pDC maturation and cytokine production. The classes of CpG ODNs have different routes of administration and produce unique downstream effects (73). In addition to CpG ODNs, a novel TLR9 agonist, IMO-2125, has also been shown to engage TLR9 leading to downstream immune signaling and suppression of A20 lymphoma and CT26 colon carcinoma tumor models in mice (80).

TLR3 and TLR8 are preferentially expressed by cDC1, which, owing to their established role in anti-tumor immunity, makes them attractive therapeutic targets (73). Polyinosinic:polycytidylic acid (Poly[I:C]) is one of the most well-known TLR3 agonists and administration of poly(I:C) is effective in inducing cDC1 maturation as well as production of IL-12, type I IFNs, and chemokines. However, as it is not well-tolerated clinically (81), variants have been developed that aim to reduce the toxicity of poly(I:C) administration. One such variant is poly-ICLC, an RNAse resistant form of poly(I:C) that leads to immune activation and some tumor responses, either alone or as an adjuvant to conventional therapies (82, 83). Poly(I:C12U), another poly(I:C) variant, introduces unpaired bases in order to increase the degradation rate of the drug in an effort to reduce adverse effects (84, 85). In addition to TLR3, cDC1 also express TLR8, which can be targeted with the TLR7/8 agonist mentioned previously, R848. Agonists of TLR8 alone are also in development. For example, VTX-2337 was shown to activate cDC1 and monocytes (86) and was well-tolerated in phase I clinical trials, although progression free survival was unchanged in a phase II trial conducted in squamous cell head and neck cancer (73, 87).

STING (stimulator of interferon genes) mediates type I IFN responses following recognition of cytosolic DNA by cGAS (cyclic GMP-AMP synthase) and production of 2′3′-cGAMP (88). Host STING is required for the induction of anti-tumor immunity, as STING-deficient mice fail to develop spontaneous immunity against immunogenic tumor lines and show reduced responses to radiation therapy (89, 90). STING knockout mice also exhibit increased susceptibility to inflammation-associated carcinogenesis following administration of AOM/DSS to induce colitis (91, 92). It is currently unclear whether STING expression by cDCs or other host cells is important for promoting an immune response, and the specifics of the tumor model and therapy being evaluated will likely impact the underlying biology. For example, blockade of CD47 promotes uptake of tumor cells by SIRPα^+^ cDC2, leading to activation of the cGAS-STING pathway (93), whereas in other tumor models it is production of 2′3′-cGAMP by tumor cells that is responsible for activation of host STING (94). Regardless, the intratumoral injection of STING agonists such as 2′3′-cGAMP and DMXAA can induce tumor rejection, both alone and in combination with other therapeutic modalities (95, 96).

Despite the pre-clinical efficacy of intratumoral injection of STING or TLR agonists, single agent efficacy in the clinic has remained elusive. This has hampered development of TLR agonists in the past, but in the age of cancer immunotherapy these are now being reevaluated as part of combinatorial therapies. For instance, a recent pre-clinical study showed that treatment with the TLR9 agonist CpG led to increased OX40 expression on CD4^+^ T cells (97). Accordingly, while intratumoral injection of CpG alone led to rejection of the directly treated tumor, the addition of an OX40 agonist antibody lead to clearance of contralateral tumors (97), and a phase I study testing this combination in non-Hodgkin lymphoma is currently underway (NCT03410901). As STING agonists have been developed more recently, these trials are already incorporating anti-PD-1 into their phase I treatment arms (e.g., NCT03010176). That said, it remains to be seen if this approach will be successful, and the development of systemic therapies targeting these pathways will be important to expand treatments beyond accessible tumors (98).

### Blocking Inhibitory Signals

Extracting murine cDCs from tumors allows them to activate and restimulate CD8^+^ T cells (4), implicating the suppressive microenvironment as a key regulator of cDC function. An alternative approach to enhance the activation state of tumor cDCs is therefore to block inhibitory pathways that reduce cDC functionality. One advantage of this approach is that it allows for systemic administration of inhibitors, as opposed to the local administration required for many immune agonists. One of the first examples of this is targeting vascular endothelial growth factor (VEGF), as VEGF inhibits DC maturation and prevents an effective anti-tumor immune response (99). VEGF inhibitors are already in clinical use to inhibit increased angiogenesis, and evidence indicates that antibodies against VEGF enhance the anti-tumor immune response by counteracting DC inhibition (100, 101). This is supported by several pre-clinical studies showing that inhibitors of VEGF increase immune function and decrease the rate of tumor growth (101–103). VEGF inhibition has also been shown to enhance DC maturation in human patients (104), suggesting that this may contribute to the efficacy of VEGF inhibitors in the clinical setting. However, it should be noted that the impact of VEGF on the vasculature and other immune populations may be more relevant to the immune impact of VEGF pathway inhibitors (105).

Another potent immunosuppressive signal in the tumor microenvironment is IL-10, which can be produced by tumor cells, macrophages, regulatory T cells, as well as other components of the stroma. Using isolated human DCs in co-culture with human melanoma cell lines, researchers have shown that IL-10 prevents DC maturation and induces a tolerogenic phenotype (106). Blockade of IL-10 in pre-clinical models, either directly or via depletion of macrophages has been shown to improve CD8^+^ T cell mediated anti-tumor immune responses in both murine and human systems (45, 106–108). At least in a mammary tumor model, this has been directly linked to the ability of IL-10 to suppress IL-12 production by cDC1s, reducing the percentage of CD8^+^ T cells that display a cytotoxic effector phenotype (45). TIM-3 expression by cDCs has also been shown to prevent response to chemotherapy in several tumor models (11, 109). How this occurs is unclear, but may relate to TIM-3 binding to high mobility box 1 protein (HMGB1) and limiting response to nucleic acids (109). Thus, while anti-TIM-3 antibodies can promote response to PD-1/L1 blocking by reducing T cell exhaustion (110, 111), TIM-3 blockade might prove efficacious even in patients with tumors that do not display substantial T cell infiltration.

Regulation of immunometabolism to increase anti-tumor immunity has been an increasing focus of cancer research. Although our understanding of basic immunometabolism is still evolving, several key insights have been made that are of relevance to tumor-associated DCs. As this has been expertly reviewed previously (99, 112), we will here highlight only two key metabolic aspects of tumor-associated DCs, and the therapeutic approaches being taken to counteract this metabolic inhibition. First, DC expression of indoleamine 2,3-dioxygenase 1 (IDO1) is thought to reduce L-tryptophan availability by converting it to L-kynurenine, leading to an increase in the suppressive capacity of regulatory T cells (113, 114). That said, IDO1 can be highly expressed by tumor cells themselves, and evidence that IDO1 expression by tumor DCs is a major mechanism of immune suppression is lacking. Several IDO1 inhibitors have also failed to demonstrate efficacy over the past few years, raising questions about the validity of this approach. Second, lipid accumulation in DCs has been shown to limit the function of DCs via interference in antigen processing and subsequent antigen presentation (115, 116). Accumulation of lipids in tumor-associated DCs is promoted by DC-specific activation of the endoplasmic reticulum (ER) stress sensor XBP1 (117). DC-specific siRNA silencing of XBP1 led to decreased lipid accumulation by DCs and enhanced immune-mediated tumor control in mouse models of ovarian cancer (117). Although further research will be required before ER stress can be effectively targeted to treat cancer, it is an active area of investigation.

### *In vivo* Expansion

Tumor cDCs are relatively infrequent in human and murine epithelial malignancies (4, 11, 33). Thus, increasing the number of intratumoral cDCs represents an alternative approach to increasing the cumulative function of the population. Rather than the injection of exogenously expanded and activated cDCs (DC vaccination; described below), it has been shown in pre-clinical studies that systemic injection of Flt3L leads to systemic expansion of the cDC1 population, increasing the number of these cells within B16 melanomas and significantly delaying tumor growth (10). This approach also showed promise in increasing both the number of cDCs in pancreatic tumors and overall control of pancreatic tumor lesions in an autochthonous disease model, highlighting the importance of DC infiltration, and expansion even in cancer types with typically low immune infiltration (118). Combined administration of Flt3L with TLR agonists, STING agonists, radiation, and/or checkpoint blockade results in additional tumor control, even in advanced tumors (7, 10, 118, 119). This approach is being tested clinically in several tumor types, including metastatic breast cancer and non-Hodgkin's lymphoma (NCT03789097, NCT01976585). The key advantage of this therapy is the potential for targeting a wider range of antigens, rather than those selected for vaccination, bypassing the need for patient-specific vaccine development. In addition, both systemic T cell activation and local T cell infiltration are enhanced by this combination, increasing the potential for synergy with other immunotherapies.

### Dendritic Cell Vaccines

**Figure 3:**
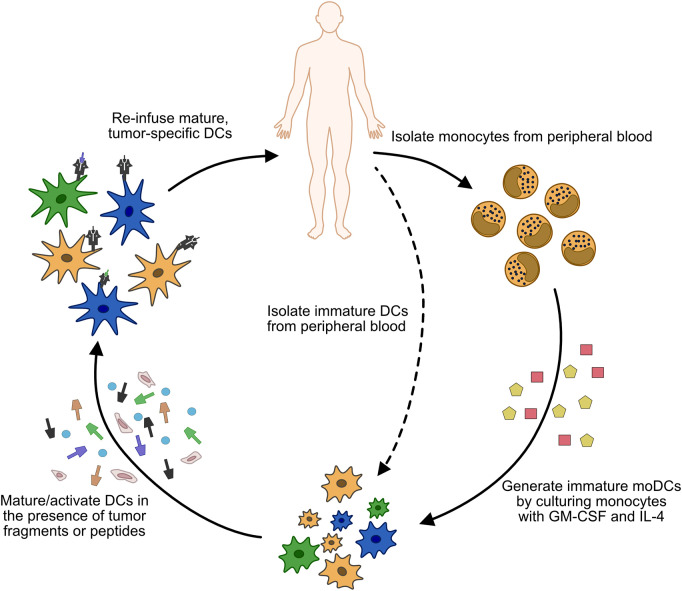
Process of generating whole cell DC vaccines. Monocytes (or less commonly, immature cDCs) are isolated from the patient's peripheral blood. In the case of monocyte isolation, immature moDCs are generated by culturing the isolated cells in GM-CSF and IL-4. Once immature DCs are obtained, they are matured/activated using a variety of cytokine cocktails, and pulsed with tumor antigen or tumor fragments. The matured DCs are then injected back into the patient, usually via subcutaneous (s.c.) or intradermal (i.d.) injections, although intravenous (i.v.) or direct injection into lymph nodes has also been used.

In contrast to *in vivo* expansion, whole-cell DC vaccines rely on exogenous maturation and/or expansion of monocyte-derived DCs or cDC precursors (Figure 3), although most trials utilize moDC due to the rarity of cDCs or pre-DCs (27). These cells are isolated from a patient's peripheral blood, loaded with tumor lysate or tumor antigens, and matured using various cytokine cocktails (120, 121). Whole cell DC vaccines are associated with limited toxicities, are therefore considered a relatively safe therapeutic approach, and are being extensively evaluated in the clinic (121, 122). Multiple vaccine formulations can lead to increased antigen-specific T cell responses. There have even been trials in AML involving the fusion of cancer cells with autologous moDCs (123). However, the presence of an immune response has not correlated with clinical efficacy (124), with response rates in general between 8 and 15% in single arm trials (122). The only whole cell DC vaccine approved by the FDA to date is sipuleucel-T, which consists of isolated PBMCs cultured with a GM-CSF/prostatic acid phosphatase fusion protein (125). This approval to treat metastatic prostate cancer was based upon a 4.1 month improvement in overall survival without an accompanying delay in disease progression (125).

Given the ability of most vaccines to induce an immune response against a specific antigen, it is unclear why vaccines have shown limited efficacy to date. One possibility is that the immunosuppressive microenvironment of the tumor blocks T cell infiltration, survival, or effector function. Several pre-clinical studies have shown that PD-1 and/or CTLA-4 blockade can improve tumor control in combination with tumor cell vaccines (126, 127). Similarly, in a mouse mammary tumor model, the efficacy of a HER2-loaded BMDC vaccine was improved by sequential anti-PD-1 therapy (128). Treatment with DC vaccines have also been shown to augment responses to standard-of-care therapy (129). Clinical trials have begun to evaluate the efficacy of combining DC vaccines with standard-of-care therapies and of vaccination with different DC subsets. In glioblastoma, a phase III clinical trial to assess the efficacy of a whole cell DC vaccine administered in combination with tumor resection, temozolomide, and radiotherapy (NCT00045968) exhibited safety and potential efficacy based on interpretation of early results (130). In contrast, a phase III trial of tumor-RNA loaded whole cell vaccines in combination with sunitinib following surgical debulking for the treatment of renal cell carcinoma (NCT01582672) was terminated early due to a lack of efficacy.

The limited efficacy of DC vaccines could also be a result of protocols that do not produce the optimal T cell response. GM-CSF maturation of PBMCs produces moDCs that are limited in their capacity to migrate to lymph nodes (131, 132), and several studies have shown endogenous DCs are actually required for T cell priming following administration of moDC vaccines (133–135). Murine cDC1s have been used in a vaccine in at least one study (136), but whether this is a viable approach in the clinic remains to be determined, particularly given the paucity of circulating, mature cDC1 in human peripheral blood (27, 137). Instead, studies have largely focused on improving baseline efficacy by assessing activation with different maturation cocktails. For many years, the “gold standard” maturation cocktail consisted of TNFα, IL-1β, IL-6, and PGE_2_ (120). However, PGE_2_ induces T regulatory cells and lowers IL-12 production, so methods of maturation which omit it are being explored. For example, an interferon cocktail along with TLR3, TLR7, and TLR8 agonists produced superior T cell mediated cytotoxicity against a breast cancer cell line (138), while the combination of TNFα, IL-1β, IFNγ, and a TLR7/8 agonist induced higher levels of the T cell chemoattractants CXCL9/10 (139). At the same time, the “gold standard” cocktail induces the highest level of DC migratory capacity (120). Given that increased DC migration to the lymph node following vaccination has been associated with increased overall survival in a small cohort of patients (140), it is unclear which approach would be better at promoting tumor control. DC migration to the lymph node can also be directly enhanced by pre-treating the injection point with DC activating agents such as tetanus toxoid and CCL3, or TLR agonists such as imiquimod or poly-ICLC (140, 141). The number of DCs injected also plays a role in achieving optimal responses, with 10^6^-10^7^ DCs per injection representing the optimal rage for efficacy (142, 143). Given the range of approaches, it remains to be seen which, if any, will produce anti-tumor responses that can induce tumor regression, either alone or in combination with other therapeutic modalities.

### Peptide/Protein Vaccines

Another possible reason for the failure of many DC vaccines may be the reliance on overexpressed or tissue-specific antigens (e.g., NY-ESO-1, MUC1, MAGEA3, MART1, HER2). In addition to their use in DC vaccines, these antigens have been fused to DC-targeting antibodies against Clec9a, DEC205, or DC-SIGN to enhance their ability to induce an immune response (122). DEC205-fused tumor-associated antigens demonstrate improved ability to induce T cell responses over administration of free antigen (144, 145). Additionally, partial clinical responses were observed following administration of DEC205-fused NY-ESO-1 and TLR agonist adjuvants in a phase I clinical trial (146). While targeting Clec9a generally induces tolerance, different adjuvants can be added in order to drive immune responses (124). For example, when combined with poly(I:C) and other adjuvants, Clec9a-fused antigens induce CD4- and CD8-mediated anti-tumor immunity (147, 148), while fusion of human IFNα to Cle9a led to an anti-tumor response that was improved by treatment with checkpoint blockade in the murine 4T1 mammary tumor model (149). Peptide fusions to antibodies against several other DC surface proteins are also in pre-clinical and clinical development (122). Given that different DC subsets can be targeted using antibodies against specifically expressed surface proteins, this represents another mechanism by which the anti-tumor immune response could be optimally shaped to induce the best outcomes for a given patient. However, one of the most recent advances in the development of cancer vaccines has been the ability to generate vaccines with patient-specific neoantigens. Although expensive and technically challenging, neoantigen vaccines are safe and able to induce strong systemic T cell responses (150, 151). More importantly, complete and durable responses have been observed in patients receiving neoantigen vaccines in combination with anti-PD-1 therapy in early phase clinical trials. Dozens of studies are now underway testing neoantigen vaccines either alone or in combination with ICB (e.g., NCT02950766, NCT03639714, NCT03953235, NCT04161755, NCT03359239).

## Concluding Remarks

Poor responses to current immunotherapies are frequently associated with tumors that have low mutational burdens or low T cell infiltration. For these patients, alternate approaches are likely necessary to elicit favorable responses on par with those observed in disease contexts such as melanoma and lung adenocarcinoma. Increasingly, the role of tumor DCs in the anti-tumor immune response is being recognized as targetable. Although single-agent therapies targeting DCs have been minimally successful, combination with standard-of-care therapies with novel immunotherapies is a promising avenue of investigation. Further research to fully understand the role of the tumor immune microenvironment as a whole is certainly warranted given the complex nature of the interactions between the tumor and immune system. A more complete understanding will hopefully lead to the development of effective therapeutic strategies that improvepatient outcomes.

## Author Contributions

All authors listed have made a substantial, direct and intellectual contribution to the work, and approved it for publication.

## Conflict of Interest

BR was supported by a sponsored research agreement with TESARO: A GSK company, has received consulting payments from Merck and Co., Inc., and has received speaking payments from Roche Farma, S.A. BR has a courtesy faculty appointment at the University of South Florida, Tampa, FL 33620. The remaining authors declare that the research was conducted in the absence of any commercial or financial relationships that could be construed as a potential conflict of interest.

## References

[1] Chen DS Mellman I. Oncology meets immunology: the cancer-immunity cycle. Immunity. (2013) 39:1–10. 10.1016/j.immuni.2013.07.012 23890059

[2] Nakayama M. Antigen presentation by MHC-Dressed cells. Front Immunol. (2014) 5:672. 10.3389/fimmu.2014.00672 25601867 PMC4283639

[3] Lin A Lore K. Granulocytes: new members of the antigen-presenting cell family. Front Immunol. (2017) 8:1781. 10.3389/fimmu.2017.01781 29321780 PMC5732227

[4] Broz ML Binnewies M Boldajipour B Nelson AE Pollack JL Erle DJ Dissecting the tumor myeloid compartment reveals rare activating antigen-presenting cells critical for T cell immunity. Cancer Cell. (2014) 26:638–52. 10.1016/j.ccell.2014.09.007 25446897 PMC4254577

[5] DeNardo DG Ruffell B. Macrophages as regulators of tumour immunity and immunotherapy. Nat Rev Immunol. (2019) 19:369–82. 10.1038/s41577-019-0127-6 30718830 PMC7339861

[6] Ruffell B Coussens LM. Macrophages and therapeutic resistance in cancer. Cancer Cell. (2015) 27:462–72. 10.1016/j.ccell.2015.02.015 25858805 PMC4400235

[7] Sanchez-Paulete AR Cueto FJ Martinez-Lopez M Labiano S Morales-Kastresana A Rodriguez-Ruiz ME. Cancer immunotherapy with immunomodulatory Anti-CD137 and Anti-PD-1 monoclonal antibodies requires BATF3-dependent dendritic cells. Cancer Discovery. (2015) 6:71–9. 10.1158/1538-7445.AM2016-4908 26493961 PMC5036540

[8] Hildner K Edelson BT Purtha WE Diamond M Matsushita H Kohyama M Batf3 deficiency reveals a critical role for CD8a+ dendritic cells in cytotoxic T cell immunity. Science. (2008) 322:1097–100. 10.1126/science.1164206 19008445 PMC2756611

[9] Roberts EW Broz ML Binnewies M Headley MB Nelson AE Wolf DM. Critical role for CD103(+)/CD141(+) dendritic cells bearing CCR7 for tumor antigen trafficking and priming of T cell immunity in melanoma. Cancer Cell. (2016) 30:324–36. 10.1016/j.ccell.2016.06.003 27424807 PMC5374862

[10] Salmon H Idoyaga J Rahman A Leboeuf M Remark R Jordan S. Expansion and activation of CD103(+) dendritic cell progenitors at the tumor site enhances tumor responses to therapeutic PD-L1 and BRAF inhibition. Immunity. (2016) 44:924–38. 10.1016/j.immuni.2016.03.012 27096321 PMC4980762

[11] de Mingo Pulido Gardner A Hiebler S Soliman H Rugo HS Krummel MF TIM-3 regulates CD103(+) dendritic cell function and response to chemotherapy in breast cancer. Cancer Cell. (2018) 33:60–74 e6. 10.1016/j.ccell.2017.11.019 29316433 PMC5764109

[12] Garris CS Arlauckas SP Kohler RH Trefny MP Garren S Piot C. Successful Anti-PD-1 cancer immunotherapy requires T cell-dendritic cell crosstalk involving the cytokines IFN-γ and IL-12. Immunity. (2018) 49:1148–61 e7. 10.1016/j.immuni.2018.09.024 30552023 PMC6301092

[13] Spranger S Dai D Horton B Gajewski TF. Tumor-residing Batf3 dendritic cells are required for effector T cell trafficking and adoptive T cell therapy. Cancer Cell. (2017) 31:711–23 e4. 10.1016/j.ccell.2017.04.003 28486109 PMC5650691

[14] Chow MT Ozga AJ Servis RL Frederick DT Lo JA Fisher DE. Intratumoral activity of the CXCR3 chemokine system is required for the efficacy of Anti-PD-1 therapy. Immunity. (2019) 50:1498–512 e5. 10.1016/j.immuni.2019.04.010 31097342 PMC6527362

[15] Gardner A Ruffell B. Dendritic cells and cancer immunity. Trends Immunol. (2016) 37:855–65. 10.1016/j.it.2016.09.006 27793569 PMC5135568

[16] Dominguez PM Ardavin C. Differentiation and function of mouse monocyte-derived dendritic cells in steady state and inflammation. Immunol Rev. (2010) 234:90–104. 10.1111/j.0105-2896.2009.00876.x 20193014

[17] Leon B Ardavin C. Monocyte-derived dendritic cells in innate and adaptive immunity. Immunol Cell Biol. (2008) 86:320–4. 10.1038/icb.2008.14 18362945

[18] Veglia F Gabrilovich DI. Dendritic cells in cancer: the role revisited. Curr Opin Immunol. (2017) 45:43–51. 10.1016/j.coi.2017.01.002 28192720 PMC5449252

[19] Collin M Bigley V. Human dendritic cell subsets: an update. Immunology. (2018) 154:3–20. 10.1111/imm.12888 29313948 PMC5904714

[20] Edelson BT Wumesh K Juang R Kohyama M Benoit LA Klekotka PA. Peripheral CD103+ dendritic cells form a unified subset developmentally related to CD8α+ conventional dendritic cells. J Exp Med. (2010) 207:823–36. 10.1084/jem.20091627 20351058 PMC2856032

[21] Sung SS Fu SM Rose CE Jr Gaskin F Ju ST Beaty SR. A major lung CD103 (αE)-β7 integrin-positive epithelial dendritic cell population expressing Langerin and tight junction proteins. J Immunol. (2006) 176:2161–72. 10.4049/jimmunol.176.4.2161 16455972

[22] Vremec D Zorbas M Scollay R Saunders D Ardavin C Wu L. The surface phenotype of dendritic cells purified from mouse thymus and spleen: investigation of the CD8 expression by a subpopulation of dendritic cells. J Exp Med. (1992) 176:47–58. 10.1084/jem.176.1.47 1613465 PMC2119290

[23] Haniffa M Shin A Bigley V McGovern N Teo P See P. Human tissues contain CD141hi cross-presenting dendritic cells with functional homology to mouse CD103+ nonlymphoid dendritic cells. Immunity. (2012) 37:60–73. 10.1016/j.immuni.2012.04.012 22795876 PMC3476529

[24] Bachem A Güttler S Hartung E Ebstein F Schaefer M Tannert A. Superior antigen cross-presentation and XCR1 expression define human CD11c+ CD141+ cells as homologues of mouse CD8+ dendritic cells. J Exp Med. (2010) 207:1273–81. 10.1084/jem.20100348 20479115 PMC2882837

[25] Jongbloed SL Kassianos AJ McDonald KJ Clark GJ Ju X Angel CE. Human CD141+ (BDCA-3)+ dendritic cells (DCs) represent a unique myeloid DC subset that cross-presents necrotic cell antigens. J Exp Med. (2010) 207:1247–60. 10.1084/jem.20092140 20479116 PMC2882828

[26] Guilliams M Ginhoux F Jakubzick C Naik SH Onai N Schraml BU. Dendritic cells, monocytes and macrophages: a unified nomenclature based on ontogeny. Nat Rev Immunol. (2014) 14:571. 10.1038/nri3712 25033907 PMC4638219

[27] Granot T Senda T Carpenter DJ Matsuoka N Weiner J Gordon CL. Dendritic cells display subset and tissue-specific maturation dynamics over human life. Immunity. (2017) 46:504–15. 10.1016/j.immuni.2017.02.019 28329707 PMC5415308

[28] Suzuki S Honma K Matsuyama T Suzuki K Toriyama K Akitoyo I. Critical roles of interferon regulatory factor 4 in CD11b^high^CD8α− dendritic cell development. Proc Natl Acad Sci USA. (2004) 101:8981–6. 10.1073/pnas.0402139101 15184678 PMC428458

[29] Vander Lugt B Khan AA Hackney JA Agrawal S Lesch J Zhou M. Transcriptional programming of dendritic cells for enhanced MHC class II antigen presentation. Nat Immunol. (2014) 15:161–7. 10.1038/ni.2795 24362890

[30] Guilliams M Dutertre CA Scott CL McGovern N Sichien D Chakarov S. Unsupervised high-dimensional analysis aligns dendritic cells across tissues and species. Immunity. (2016) 45:669–84. 10.1016/j.immuni.2016.08.015 27637149 PMC5040826

[31] Zilionis R Engblom C Pfirschke C Savova V Zemmour D Saatcioglu HD. Single-cell transcriptomics of human and mouse lung cancers reveals conserved myeloid populations across individuals and species. Immunity. (2019) 50:1317–34.e10. 10.1016/j.immuni.2019.03.009 30979687 PMC6620049

[32] Sittig SP Bakdash G Weiden J Skold AE Tel J Figdor CG. A comparative study of the T cell stimulatory and polarizing capacity of human primary blood dendritic cell subsets. Mediators Inflamm. (2016) 2016:3605643. 10.1155/2016/3605643 27057096 PMC4761397

[33] Binnewies M Mujal AM Pollack JL Combes AJ Hardison EA Barry KC. Unleashing Type-2 dendritic cells to drive protective antitumor CD4(+) T cell immunity. Cell. (2019) 177:556–71.e16. 10.1016/j.cell.2019.02.005 30955881 PMC6954108

[34] Fuertes MB Kacha AK Kline J Woo SR Kranz DM Murphy KM Host type I IFN signals are required for antitumor CD8+ T cell responses through CD8α+ dendritic cells. J Exp Med. (2011) 208:2005–16. 10.1084/jem.20101159 21930765 PMC3182064

[35] Spranger S Bao R Gajewski TF. Melanoma-intrinsic β-catenin signalling prevents anti-tumour immunity. Nature. (2015) 523:231–5. 10.1038/nature14404 25970248

[36] Barry KC Hsu J Broz ML Cueto FJ Binnewies M Combes AJ. A natural killer-dendritic cell axis defines checkpoint therapy-responsive tumor microenvironments. Nat Med. (2018) 24:1178–91. 10.1038/s41591-018-0085-8 29942093 PMC6475503

[37] Allan RS Waithman J Bedoui S Jones CM Villadangos JA Zhan Y. Migratory dendritic cells transfer antigen to a lymph node-resident dendritic cell population for efficient CTL priming. Immunity. (2006) 25:153–62. 10.1016/j.immuni.2006.04.017 16860764

[38] Itano AA McSorley SJ Reinhardt RL Ehst BD Ingulli E Rudensky AY. Distinct dendritic cell populations sequentially present antigen to CD4 T cells and stimulate different aspects of cell-mediated immunity. Immunity. (2003) 19:47–57. 10.1016/S1074-7613(03)00175-4 12871638

[39] Theisen DJ Davidson JT Briseño CG Gargaro M Lauron EJ Wang Q. WDFY4 is required for cross-presentation in response to viral and tumor antigens. Science. (2018) 362:694–9. 10.1126/science.aat5030 30409884 PMC6655551

[40] Diamond MS Kinder M Matsushita H Mashayekhi M Dunn GP Archambault JM. Type I interferon is selectively required by dendritic cells for immune rejection of tumors. J Exp Med. (2011) 208:1989–2003. 10.1084/jem.20101158 21930769 PMC3182061

[41] Eisenbarth S. Dendritic cell subsets in T cell programming: location dictates function. Nat Rev Immunol. (2018) 19:89–103. 10.1038/s41577-018-0088-1 30464294 PMC7755085

[42] Maurice NJ McElrath MJ Andersen-Nissen E Frahm N Prlic M. CXCR3 enables recruitment and site-specific bystander activation of memory CD8+ T cells. Nat Commun. (2019) 10:1–15. 10.1038/s41467-019-12980-2 31676770 PMC6825240

[43] Natsuaki Y Egawa G Nakamizo S Ono S Hanakawa S Okada T. Perivascular leukocyte clusters are essential for efficient activation of effector T cells in the skin. Nat Immunol. (2014) 15:1064–9. 10.1038/ni.2992 25240383

[44] Calabro S Liu D Gallman A Nascimento MS Yu Z Zhang T-T. Differential intrasplenic migration of dendritic cell subsets tailors adaptive immunity. Cell Rep. (2016) 16:2472–85. 10.1016/j.celrep.2016.07.076 27545885 PMC6323650

[45] Ruffell B Chang-Strachan D Chan V Rosenbusch A Ho CM Pryer N. Macrophage IL-10 blocks CD8+ T cell-dependent responses to chemotherapy by suppressing IL-12 expression in intratumoral dendritic cells. Cancer Cell. (2014) 26:623–37. 10.1016/j.ccell.2014.09.006 25446896 PMC4254570

[46] Maier B Leader AM Chen ST Tung N Chang C LeBerichel J. A conserved dendritic-cell regulatory program limits antitumour immunity. Nature. (2020) 580:257–62. 10.1038/s41586-020-2134-y 32269339 PMC7787191

[47] Bottcher JP Bonavita E Chakravarty P Blees H Cabeza-Cabrerizo M Sammicheli S. NK cells stimulate recruitment of cDC1 into the tumor microenvironment promoting cancer immune control. Cell. (2018) 172:1022–37 e14. 10.1016/j.cell.2018.01.004 29429633 PMC5847168

[48] Mittal D Vijayan D Putz EM Aguilera AR Markey KA Straube J. Interleukin-12 from CD103+ Batf3-dependent dendritic cells required for NK-cell suppression of metastasis. Cancer Immunol Res. (2017) 5:1098–108. 10.1158/2326-6066.CIR-17-0341 29070650

[49] Zhang Q He Y Luo N Patel SJ Han Y Gao R. Landscape and dynamics of single immune cells in hepatocellular carcinoma. Cell. (2019) 179:829–45. e20. 10.1016/j.cell.2019.10.003 31675496

[50] Fridman WH Pages F Sautes-Fridman C Galon J. The immune contexture in human tumours: impact on clinical outcome. Nat Rev Cancer. (2012) 12:298–306. 10.1038/nrc3245 22419253

[51] Janssen EM Lemmens EE Wolfe T Christen U von Herrath MG Schoenberger SP. CD4+ T cells are required for secondary expansion and memory in CD8+ T lymphocytes. Nature. (2003) 421:852. 10.1038/nature01441 12594515

[52] Matloubian M Concepcion RJ Ahmed R. CD4+ T cells are required to sustain CD8+ cytotoxic T-cell responses during chronic viral infection. J Virol. (1994) 68:8056–63. 10.1128/JVI.68.12.8056-8063.1994 7966595 PMC237269

[53] Shedlock DJ Shen H. Requirement for CD4 T cell help in generating functional CD8 T cell memory. Science. (2003) 300:337–9. 10.1126/science.1082305 12690201

[54] Sun JC Bevan MJ. Defective CD8 T cell memory following acute infection without CD4 T cell help. Science. (2003) 300:339–42. 10.1126/science.1083317 12690202 PMC2778341

[55] Chung AS Wu X Zhuang G Ngu H Kasman I Zhang J. An interleukin-17-mediated paracrine network promotes tumor resistance to anti-angiogenic therapy. Nat Med. (2013) 19:1114–23. 10.1038/nm.3291 23913124

[56] Zanetti M. Tapping CD4 T cells for cancer immunotherapy: the choice of personalized genomics. J Immunol. (2015) 194:2049–56. 10.4049/jimmunol.1402669 25710958

[57] Brown CC Gudjonson H Pritykin Y Deep D Lavallée V-P Mendoza A. Transcriptional basis of mouse and human dendritic cell heterogeneity. Cell. (2019) 179:846–63. e24. 10.1016/j.cell.2019.09.035 31668803 PMC6838684

[58] Villadangos JA Young L. Antigen-presentation properties of plasmacytoid dendritic cells. Immunity. (2008) 29:352–61. 10.1016/j.immuni.2008.09.002 18799143

[59] Rodrigues PF Alberti-Servera L Eremin A Grajales-Reyes GE Ivanek R Tussiwand R. Distinct progenitor lineages contribute to the heterogeneity of plasmacytoid dendritic cells. Nat Immunol. (2018) 19:711. 10.1038/s41590-018-0136-9 29925996 PMC7614340

[60] Le Mercier I Poujol D Sanlaville A Sisirak V Gobert M Durand I. Tumor promotion by intratumoral plasmacytoid dendritic cells is reversed by TLR7 ligand treatment. Cancer Res. (2013) 73:4629–40. 10.1158/0008-5472.CAN-12-3058 23722543

[61] Kranz LM Diken M Haas H Kreiter S Loquai C Reuter KC. Systemic RNA delivery to dendritic cells exploits antiviral defence for cancer immunotherapy. Nature. (2016) 534:396–401. 10.1038/nature18300 27281205

[62] Treilleux I Blay J-Y Bendriss-Vermare N Ray-Coquard I Bachelot T Guastalla J-P. Dendritic cell infiltration and prognosis of early stage breast cancer. Clin Cancer Res. (2004) 10:7466–74. 10.1158/1078-0432.CCR-04-0684 15569976

[63] Zou W Machelon V Coulomb-L'Hermin A Borvak J Nome F Isaeva T. Stromal-derived factor-1 in human tumors recruits and alters the function of plasmacytoid precursor dendritic cells. Nat Med. (2001) 7:1339. 10.1038/nm1201-1339 11726975

[64] Alcántara-Hernández M Leylek R Wagar LE Engleman EG Keler T Marinkovich MP. High-dimensional phenotypic mapping of human dendritic cells reveals interindividual variation and tissue specialization. Immunity. (2017) 47:1037–50. e6. 10.1016/j.immuni.2017.11.001 29221729 PMC5738280

[65] Villani A-C Satija R Reynolds G Sarkizova S Shekhar K Fletcher J. Single-cell RNA-seq reveals new types of human blood dendritic cells, monocytes, and progenitors. Science. (2017) 356:eaah4573. 10.1126/science.aah4573 28428369 PMC5775029

[66] See P Dutertre C-A Chen J Günther P McGovern N Irac SE. Mapping the human DC lineage through the integration of high-dimensional techniques. Science. (2017) 356:eaag3009. 10.1126/science.aag3009 28473638 PMC7611082

[67] Guilliams M Bruhns P Saeys Y Hammad H Lambrecht BN. The function of Fcγ receptors in dendritic cells and macrophages. Nat Rev Immunol. (2014) 14:94–108. 10.1038/nri3582 24445665

[68] Gilfillan CB Kuhn S Baey C Hyde EJ Yang J Ruedl C Clec9A+ dendritic cells are not essential for antitumor CD8+ T cell responses induced by Poly I: C immunotherapy. J Immunol. (2018) 200:2978–86. 10.4049/jimmunol.1701593 29507107

[69] Marigo I Zilio S Desantis G Mlecnik B Agnellini AH Ugel S T cell cancer therapy requires CD40-CD40L activation of tumor necrosis factor and inducible nitric-oxide-synthase-producing dendritic cells. Cancer Cell. (2016) 30:377–90. 10.1016/j.ccell.2016.08.004 27622331 PMC5023283

[70] Sharma MD Rodriguez PC Koehn BH Baban B Cui Y Guo G Activation of p53 in myeloid precursor cells controls differentiation into immunogenic Ly6c+CD103+ monocytic cells in tumors. Immunity. (2018) 48:91–106.e6. 10.1016/j.immuni.2017.12.014 29343444 PMC6005382

[71] Betts BC Locke FL Sagatys EM Pidala J Walton K Menges M. Inhibition of human dendritic cell ER stress response reduces T cell alloreactivity yet spares donor anti-tumor immunity. Front Immunol. (2018) 9:2887. 10.3389/fimmu.2018.02887 30574153 PMC6291501

[72] Schutt SD Wu Y Tang C-HA Bastian D Nguyen H Sofi MH. Inhibition of the IRE-1α/XBP-1 pathway prevents chronic GVHD and preserves the GVL effect in mice. Blood Adv. (2018) 2:414–27. 10.1182/bloodadvances.2017009068 29483082 PMC5858472

[73] Chiang CL Kandalaft LE. *In vivo* cancer vaccination: which dendritic cells to target and how? Cancer Treat Rev. (2018) 71:88–101. 10.1016/j.ctrv.2018.10.012 30390423 PMC6295330

[74] Pathria P Louis TL Varner JA. Targeting tumor-associated macrophages in cancer. Trends Immunol. (2019) 40:310–27. 10.1016/j.it.2019.02.003 30890304

[75] Adams S Kozhaya L Martiniuk F Meng T-C Chiriboga L Liebes L. Topical TLR7 agonist imiquimod can induce immune-mediated rejection of skin metastases in patients with breast cancer. Clin Cancer Res. (2012) 18:6748–57. 10.1158/1078-0432.CCR-12-1149 22767669 PMC3580198

[76] Salazar LG Lu H Reichow JL Childs JS Coveler AL Higgins DM. Topical imiquimod plus nab-paclitaxel for breast cancer cutaneous metastases: a phase 2 clinical trial. JAMA Oncol. (2017) 3:969–73. 10.1001/jamaoncol.2016.6007 28114604 PMC5824239

[77] Adams S O'Neill DW Nonaka D Hardin E Chiriboga L Siu K. Immunization of malignant melanoma patients with full-length NY-ESO-1 protein using TLR7 agonist imiquimod as vaccine adjuvant. J Immunol. (2008) 181:776–84. 10.4049/jimmunol.181.1.776 18566444 PMC2583094

[78] Dudek AZ Yunis C Harrison LI Kumar S Hawkinson R Cooley S. First in human phase I trial of 852A, a novel systemic toll-like receptor 7 agonist, to activate innate immune responses in patients with advanced cancer. Clin Cancer Res. (2007) 13:7119–25. 10.1158/1078-0432.CCR-07-1443 18056192

[79] Harrison LI Astry C Kumar S Yunis C. Pharmacokinetics of 852A, an imidazoquinoline Toll-like receptor 7-specific agonist, following intravenous, subcutaneous, and oral administrations in humans. J Clin Pharmacol. (2007) 47:962–9. 10.1177/0091270007303766 17660481

[80] Wang D Jiang W Zhu F Mao X Agrawal S. Modulation of the tumor microenvironment by intratumoral administration of IMO-2125, a novel TLR9 agonist, for cancer immunotherapy. Int J Oncol. (2018) 53:1193–203. 10.3892/ijo.2018.4456 29956749

[81] Robinson RA DeVita VT Levy HB Baron S Hubbard SP Levine AS A Phase I–II trial of multiple-dose polyriboinosinic-polyribocytidylic acid in patients with leukemia or solid tumors. J Natl Cancer Inst. (1976) 57:599–602. 10.1093/jnci/57.3.599 978771

[82] Andres MS Hilton BL Steven O Meir K Barbara S Hernando M Long-term treatment of malignant gliomas with intramuscularly administered polyinosinic-polycytidylic acid stabilized with polylysine and carboxymethylcellulose: an open pilot study. Neurosurgery. (1996) 38:1096–104. 10.1097/00006123-199606000-00006 8727138

[83] Caskey M Lefebvre F Filali-Mouhim A Cameron MJ Goulet J-P Haddad EK Breton G Trumpfheller C Pollak S Shimeliovich I. Synthetic double-stranded RNA induces innate immune responses similar to a live viral vaccine in humans. J Exp Med. (2011) 208:2357–66. 10.1084/jem.20111171 22065672 PMC3256967

[84] Navabi H Jasani B Reece A Clayton A Tabi Z Donninger C A clinical grade poly I: C-analogue (Ampligen®) promotes optimal DC maturation and Th1-type T cell responses of healthy donors and cancer patients *in vitro*. Vaccine. (2009) 27:107–15. 10.1016/j.vaccine.2008.10.024 18977262

[85] Gowen BB Wong M-H Jung K-H Sanders AB Mitchell WM Alexopoulou L TLR3 is essential for the induction of protective immunity against Punta Toro Virus infection by the double-stranded RNA (dsRNA), poly (I: C12U), but not Poly (I: C): differential recognition of synthetic dsRNA molecules. J Immunol. (2007) 178:5200–8. 10.4049/jimmunol.178.8.5200 17404303

[86] Lu H Dietsch GN Matthews M-AH Yang Y Ghanekar S Inokuma M. VTX-2337 is a novel TLR8 agonist that activates NK cells and augments ADCC. Clin Cancer Res. (2012) 18:499–509. 10.1158/1078-0432.CCR-11-1625 22128302

[87] Chow LQ Morishima C Eaton KD Baik CS Goulart BH Anderson LN Phase Ib trial of the toll-like receptor 8 agonist, motolimod (VTX-2337), combined with cetuximab in patients with recurrent or metastatic SCCHN. Clin Cancer Res. (2017) 23:2442–50. 10.1158/1078-0432.CCR-16-1934 27810904

[88] Kwon J Bakhoum SF. The cytosolic DNA-sensing cGAS-STING pathway in cancer. Cancer Discovery. (2020) 10:26–39. 10.1158/2159-8290.CD-19-0761 31852718 PMC7151642

[89] Woo SR Fuertes MB Corrales L Spranger S Furdyna MJ Leung MY. STING-dependent cytosolic DNA sensing mediates innate immune recognition of immunogenic tumors. Immunity. (2014) 41:830–42. 10.1016/j.immuni.2014.10.017 25517615 PMC4384884

[90] Deng L Liang H Xu M Yang X Burnette B Arina A. STING-dependent cytosolic DNA sensing promotes radiation-induced Type I interferon-dependent antitumor immunity in immunogenic tumors. Immunity. (2014) 41:843–52. 10.1016/j.immuni.2014.10.019 25517616 PMC5155593

[91] Ahn J Konno H Barber GN. Diverse roles of STING-dependent signaling on the development of cancer. Oncogene. (2015) 34:5302. 10.1038/onc.2014.457 25639870 PMC4998969

[92] Zhu Q Man SM Gurung P Liu Z Vogel P Lamkanfi M. Cutting edge: STING mediates protection against colorectal tumorigenesis by governing the magnitude of intestinal inflammation. J Immunol. (2014) 193:4779–82. 10.4049/jimmunol.1402051 25320273 PMC4308418

[93] Xu MM Pu Y Han D Shi Y Cao X Liang H Dendritic cells but not macrophages sense tumor mitochondrial DNA for cross-priming through signal regulatory protein α signaling. Immunity. (2017) 47:363–73 e5. 10.1016/j.immuni.2017.07.016 28801234 PMC5564225

[94] Marcus A Mao AJ Lensink-Vasan M Wang L Vance RE Raulet DH. Tumor-derived cGAMP triggers a STING-mediated interferon response in non-tumor cells to activate the NK cell response. Immunity. (2018) 49:754–63 e4. 10.1016/j.immuni.2018.09.016 30332631 PMC6488306

[95] Corrales L Glickman LH McWhirter SM Kanne DB Sivick KE Katibah GE. Direct activation of STING in the tumor microenvironment leads to potent and systemic tumor regression and immunity. Cell Rep. (2015) 11:1018–30. 10.1016/j.celrep.2015.04.031 25959818 PMC4440852

[96] Baird JR Friedman D Cottam B Dubensky TW Kanne DB Bambina S. Radiotherapy combined with novel STING-targeting oligonucleotides results in regression of established tumors. Cancer Res. (2016) 76:50–61. 10.1158/0008-5472.CAN-14-3619 26567136 PMC4703500

[97] Sagiv-Barfi I Czerwinski DK Levy S Alam IS Mayer AT Gambhir SS. Eradication of spontaneous malignancy by local immunotherapy. Sci Transl Med. (2018) 10:eaan4488. 10.1126/scitranslmed.aan4488 29386357 PMC5997264

[98] Ramanjulu JM Pesiridis GS Yang J Concha N Singhaus R Zhang S-Y. Design of amidobenzimidazole STING receptor agonists with systemic activity. Nature. (2018) 564:439–443. 10.1038/s41586-018-0705-y 30405246

[99] Giovanelli P Sandoval TA Cubillos-Ruiz JR. Dendritic cell metabolism and function in tumors. Trends Immunol. (2019) 40:699–718. 10.1016/j.it.2019.06.004 31301952

[100] Gabrilovich DI Chen HL Girgis KR Cunningham HT Meny GM Nadaf S. Production of vascular endothelial growth factor by human tumors inhibits the functional maturation of dendritic cells. Nat Med. (1996) 2:1096–103. 10.1038/nm1096-1096 8837607

[101] Gabrilovich DI Ishida T Nadaf S Ohm JE Carbone DP. Antibodies to vascular endothelial growth factor enhance the efficacy of cancer immunotherapy by improving endogenous dendritic cell function. Clin Cancer Res. (1999) 5:2963–70. 10537366

[102] Ohm JE Gabrilovich DI Sempowski GD Kisseleva E Parman KS Nadaf S. VEGF inhibits T-cell development and may contribute to tumor-induced immune suppression. Blood. (2003) 101:4878–86. 10.1182/blood-2002-07-1956 12586633

[103] Alfaro C Suarez N Gonzalez A Solano S Erro L Dubrot J. Influence of bevacizumab, sunitinib and sorafenib as single agents or in combination on the inhibitory effects of VEGF on human dendritic cell differentiation from monocytes. Br J Cancer. (2009) 100:1111. 10.1038/sj.bjc.6604965 19277038 PMC2670006

[104] Osada T Chong G Tansik R Hong T Spector N Kumar R. The effect of anti-VEGF therapy on immature myeloid cell and dendritic cells in cancer patients. Cancer Immunol Immunother. (2008) 57:1115–24. 10.1007/s00262-007-0441-x 18193223 PMC4110970

[105] Schaaf MB Garg AD Agostinis P. Defining the role of the tumor vasculature in antitumor immunity and immunotherapy. Cell Death Dis. (2018) 9:115. 10.1038/s41419-017-0061-0 29371595 PMC5833710

[106] Steinbrink K Jonuleit H Müller G Schuler G Knop J Enk AH. Interleukin-10–treated human dendritic cells induce a melanoma-antigen–specific anergy in CD8+ T cells resulting in a failure to lyse tumor cells. Blood. (1999) 93:1634–42. 10.1182/blood.V93.5.1634.405k11_1634_1642 10029592

[107] Vicari AP Chiodoni C Vaure C Aït-Yahia S Dercamp C Matsos F. Reversal of tumor-induced dendritic cell paralysis by CpG immunostimulatory oligonucleotide and anti–interleukin 10 receptor antibody. J Exp Med. (2002) 196:541–9. 10.1084/jem.20020732 12186845 PMC2196048

[108] Steinbrink K Wölfl M Jonuleit H Knop J Enk AH. Induction of tolerance by IL-10-treated dendritic cells. J Immunol. (1997) 159:4772–80. 9366401

[109] Chiba S Baghdadi M Akiba H Yoshiyama H Kinoshita I Dosaka-Akita H. Tumor-infiltrating DCs suppress nucleic acid-mediated innate immune responses through interactions between the receptor TIM-3 and the alarmin HMGB1. Nat Immunol. (2012) 13:832–42. 10.1038/ni.2376 22842346 PMC3622453

[110] Ngiow SF von Scheidt B Akiba H Yagita H Teng MW Smyth MJ. Anti-TIM3 antibody promotes T cell IFN-gamma-mediated antitumor immunity and suppresses established tumors. Cancer Res. (2011) 71:3540–51. 10.1158/0008-5472.CAN-11-0096 21430066

[111] Sakuishi K Apetoh L Sullivan JM Blazar BR Kuchroo VK Anderson AC. Targeting Tim-3 and PD-1 pathways to reverse T cell exhaustion and restore anti-tumor immunity. J Exp Med. (2010) 207:2187–94. 10.1084/jem.20100643 20819927 PMC2947065

[112] Pearce EJ Everts B. Dendritic cell metabolism. Nat Rev Immunol. (2015) 15:18–29. 10.1038/nri3771 25534620 PMC4495583

[113] Munn DH Mellor AL. IDO in the tumor microenvironment: inflammation, counter-regulation, and tolerance. Trends Immunol. (2016) 37:193–207. 10.1016/j.it.2016.01.002 26839260 PMC4916957

[114] Matteoli G Mazzini E Iliev ID Mileti E Fallarino F Puccetti P. Gut CD103+ dendritic cells express indoleamine 2, 3-dioxygenase which influences T regulatory/T effector cell balance and oral tolerance induction. Gut. (2010) 59:595–604. 10.1136/gut.2009.185108 20427394

[115] Herber DL Cao W Nefedova Y Novitskiy SV Nagaraj S Tyurin VA. Lipid accumulation and dendritic cell dysfunction in cancer. Nat Med. (2010) 16:880–6. 10.1038/nm.2172 20622859 PMC2917488

[116] Veglia F Tyurin VA Mohammadyani D Blasi M Duperret EK Donthireddy L. Lipid bodies containing oxidatively truncated lipids block antigen cross-presentation by dendritic cells in cancer. Nat Commun. (2017) 8:2122. 10.1038/s41467-017-02186-9 29242535 PMC5730553

[117] Cubillos-Ruiz JR Silberman PC Rutkowski MR Chopra S Perales-Puchalt A Song M. ER stress sensor XBP1 controls anti-tumor immunity by disrupting dendritic cell homeostasis. Cell. (2015) 161:1527–38. 10.1016/j.cell.2015.05.025 26073941 PMC4580135

[118] Hegde S Krisnawan VE Herzog BH Zuo C Breden MA Knolhoff BL. Dendritic cell paucity leads to dysfunctional immune surveillance in pancreatic cancer. Cancer Cell. (2020) 37:289–307. e9. 10.1016/j.ccell.2020.02.008 32183949 PMC7181337

[119] Hammerich L Marron TU Upadhyay R Svensson-Arvelund J Dhainaut M Hussein S. Systemic clinical tumor regressions and potentiation of PD1 blockade with in situ vaccination. Nat Med. (2019) 25:814–24. 10.1038/s41591-019-0410-x 30962585

[120] Massa C Thomas C Wang E Marincola F Seliger B. Different maturation cocktails provide dendritic cells with different chemoattractive properties. J Transl Med. (2015) 13:175. 10.1186/s12967-015-0528-7 26695182 PMC4467838

[121] Bol KF Schreibelt G Gerritsen WR De Vries IJ Figdor CG. Dendritic cell–based immunotherapy: state of the art and beyond. Clin Cancer Res. (2016) 22:1897–906. 10.1158/1078-0432.CCR-15-1399 27084743

[122] Wculek SK Cueto FJ Mujal AM Melero I Krummel MF Sancho D. Dendritic cells in cancer immunology and immunotherapy. Nat Rev. Immunol. (2019) 20:7–24. 10.1038/s41577-019-0210-z 31467405

[123] Rosenblatt J Stone RM Uhl L Neuberg D Joyce R Levine JD. Individualized vaccination of AML patients in remission is associated with induction of antileukemia immunity and prolonged remissions. Sci Transl Med. (2016) 8:368ra171–368ra171. 10.1126/scitranslmed.aag1298 27928025 PMC5800949

[124] Sabado RL Balan S Bhardwaj N. Dendritic cell-based immunotherapy. Cell Res. (2017) 27:74. 10.1038/cr.2016.157 28025976 PMC5223236

[125] Kantoff PW Higano CS Shore ND Berger ER Small EJ Penson DF. Sipuleucel-T immunotherapy for castration-resistant prostate cancer. N Engl J Med. (2010) 363:411–22. 10.1056/NEJMoa1001294 20818862

[126] Duraiswamy J Kaluza KM Freeman GJ Coukos G. Dual blockade of PD-1 and CTLA-4 combined with tumor vaccine effectively restores T-cell rejection function in tumors. Cancer Res. (2013) 73:3591–603. 10.1158/0008-5472.CAN-12-4100 23633484 PMC3686913

[127] Curran MA Montalvo W Yagita H Allison JP. PD-1 and CTLA-4 combination blockade expands infiltrating T cells and reduces regulatory T and myeloid cells within B16 melanoma tumors. Proc Natl Acad Sci USA. (2010) 107:4275–80. 10.1073/pnas.0915174107 20160101 PMC2840093

[128] Kodumudi KN Ramamoorthi G Snyder C Basu A Jia Y Awshah S. Sequential anti-PD1 therapy following dendritic cell vaccination improves survival in a HER2 mammary carcinoma model and identifies a critical role for CD4 T cells in mediating the response. Front Immunol. (2019) 10:1939. 10.3389/fimmu.2019.01939 31475002 PMC6702967

[129] Soliman H Khambati F Han HS Ismail-Khan R Bui MM Sullivan DM. A phase-1/2 study of adenovirus-p53 transduced dendritic cell vaccine in combination with indoximod in metastatic solid tumors and invasive breast cancer. Oncotarget. (2018) 9:10110. 10.18632/oncotarget.24118 29515795 PMC5839376

[130] Liau LM Ashkan K Tran DD Campian JL Trusheim JE Cobbs CS First results on survival from a large Phase 3 clinical trial of an autologous dendritic cell vaccine in newly diagnosed glioblastoma. J Transl Med. (2018) 16:142 10.1186/s12967-018-1507-6 29843811 PMC5975654

[131] Morse MA Coleman RE Akabani G Niehaus N Coleman D Lyerly HK. Migration of human dendritic cells after injection in patients with metastatic malignancies. Cancer Res. (1999) 59:56–8. 9892184

[132] De Vries IJ Krooshoop DJ Scharenborg NM Lesterhuis WJ Diepstra JH Van Muijen GN. Effective migration of antigen-pulsed dendritic cells to lymph nodes in melanoma patients is determined by their maturation state. Cancer Res. (2003) 63:12–7. 12517769

[133] Kleindienst P Brocker T. Endogenous dendritic cells are required for amplification of T cell responses induced by dendritic cell vaccines *in vivo*. J Immunol. (2003) 170:2817–23. 10.4049/jimmunol.170.6.2817 12626531

[134] Yewdall AW Drutman SB Jinwala F Bahjat KS Bhardwaj N. CD8+ T cell priming by dendritic cell vaccines requires antigen transfer to endogenous antigen presenting cells. PLoS ONE. (2010) 5:e11144. 10.1371/journal.pone.0011144 20585396 PMC2886840

[135] Petersen TR Sika-Paotonu D Knight DA Simkins HM Hermans IF. Exploiting the role of endogenous lymphoid-resident dendritic cells in the priming of NKT cells and CD8+ T cells to dendritic cell-based vaccines. PLoS ONE. (2011) 6:e17657. 10.1371/journal.pone.0017657 21483862 PMC3069042

[136] Wculek SK Amores-Iniesta J Conde-Garrosa R Khouili SC Melero I Sancho D. Effective cancer immunotherapy by natural mouse conventional type-1 dendritic cells bearing dead tumor antigen. J Immunother Cancer. (2019) 7:100. 10.1186/s40425-019-0565-5 30961656 PMC6454603

[137] Perez CR De Palma M. Engineering dendritic cell vaccines to improve cancer immunotherapy. Nat Commun. (2019) 10:5408. 10.1038/s41467-019-13368-y 31776331 PMC6881351

[138] Tomasicchio M Semple L Esmail A Meldau R Randall P Pooran A. An autologous dendritic cell vaccine polarizes a Th-1 response which is tumoricidal to patient-derived breast cancer cells. Cancer Immunol Immunother. (2019) 68:71–83. 10.1007/s00262-018-2238-5 30283982 PMC6326986

[139] Massa C Seliger B. Fast dendritic cells stimulated with alternative maturation mixtures induce polyfunctional and long-lasting activation of innate and adaptive effector cells with tumor-killing capabilities. J Immunol. (2013) 190:3328–37. 10.4049/jimmunol.1202024 23447683

[140] Mitchell DA Batich KA Gunn MD Huang MN Sanchez-Perez L Nair SK. Tetanus toxoid and CCL3 improve dendritic cell vaccines in mice and glioblastoma patients. Nature. (2015) 519:366–9. 10.1038/nature14320 25762141 PMC4510871

[141] Prins RM Soto H Konkankit V Odesa SK Eskin A Yong WH. Gene expression profile correlates with T-cell infiltration and relative survival in glioblastoma patients vaccinated with dendritic cell immunotherapy. Clin Cancer Res. (2011) 17:1603–15. 10.1158/1078-0432.CCR-10-2563 21135147 PMC3071163

[142] Aarntzen EH Srinivas M Bonetto F Cruz LJ Verdijk P Schreibelt G. Targeting of 111In-labeled dendritic cell human vaccines improved by reducing number of cells. Clin Cancer Res. (2013) 19:1525–33. 10.1158/1078-0432.CCR-12-1879 23382117

[143] Martín-Fontecha A Sebastiani S Höpken UE Uguccioni M Lipp M Lanzavecchia A. Regulation of dendritic cell migration to the draining lymph node: impact on T lymphocyte traffic and priming. J Exp Med. (2003) 198:615–21. 10.1084/jem.20030448 12925677 PMC2194169

[144] Birkholz K Schwenkert M Kellner C Gross S Fey G Schuler-Thurner B. Targeting of DEC-205 on human dendritic cells results in efficient MHC class II–restricted antigen presentation. Blood. (2010) 116:2277–85. 10.1182/blood-2010-02-268425 20566893

[145] Tsuji T Matsuzaki J Kelly MP Ramakrishna V Vitale L He L-Z. Antibody-targeted NY-ESO-1 to mannose receptor or DEC-205 *in vitro* elicits dual human CD8+ and CD4+ T cell responses with broad antigen specificity. J Immunol. (2011) 186:1218–27. 10.4049/jimmunol.1000808 21149605

[146] Dhodapkar MV Sznol M Zhao B Wang D Carvajal RD Keohan ML. Induction of antigen-specific immunity with a vaccine targeting NY-ESO-1 to the dendritic cell receptor DEC-205. Sci Transl Med. (2014) 6:232ra51. 10.1126/scitranslmed.3008068 24739759 PMC6151129

[147] Sancho D Mourão-Sá D Joffre OP Schulz O Rogers NC Pennington DJ. Tumor therapy in mice via antigen targeting to a novel, DC-restricted C-type lectin. J Clin Invest. (2008) 118:2098–110. 10.1172/JCI34584 18497879 PMC2391066

[148] Joffre OP Sancho D Zelenay S Keller AM C Reis e Sousa. Efficient and versatile manipulation of the peripheral CD4+ T-cell compartment by antigen targeting to DNGR-1/CLEC9A. Eur J Immunol. (2010) 40:1255–65. 10.1002/eji.201040419 20333625 PMC3064981

[149] Cauwels A Van Lint S Paul F Garcin G De Koker S Van Parys A. Delivering type I interferon to dendritic cells empowers tumor eradication and immune combination treatments. Cancer Res. (2018) 78:463–74. 10.1158/0008-5472.CAN-17-1980 29187401

[150] Ott PA Hu Z Keskin DB Shukla SA Sun J Bozym DJ. An immunogenic personal neoantigen vaccine for patients with melanoma. Nature. (2017) 547:217–21. 10.1038/nature22991 28678778 PMC5577644

[151] Sahin U Derhovanessian E Miller M Kloke BP Simon P Lower M. Personalized RNA mutanome vaccines mobilize poly-specific therapeutic immunity against cancer. Nature. (2017) 547:222–6. 10.1038/nature23003 28678784

